# Supplementary material for: Shared Treg dysregulation underlies psoriasis vulgaris and atopic dermatitis through the PHF19-PRC2 epigenetic axis
Source: Front Immunol. 2026 Aug 20;17:1932788. doi: 10.3389/fimmu.2026.1932788 (PMC13538097; doi:10.3389/fimmu.2026.1932788)
Supplement: Supplementary file 3 [file DataSheet3.zip › Additional files/Additional file 2/16.ETFB.forest.pdf]

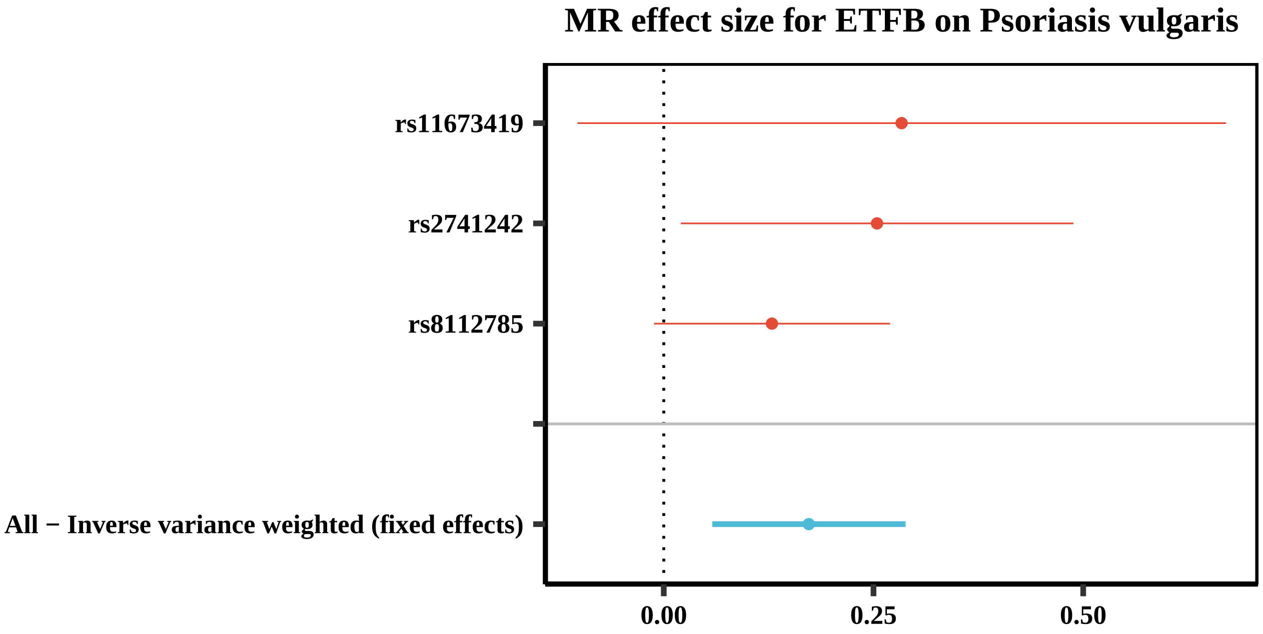
MR effect size for ETFB on Psoriasis vulgaris
rs11673419
rs2741242
rs8112785
All − Inverse variance weighted (fixed effects)
0.00
0.25
0.50
